# ATTITUDES TOWARD AGING AMONG COLLEGE STUDENTS: RESULTS FROM THE INTERGENERATIONAL CONNECTIONS PROJECT

**DOI:** 10.1093/geroni/igac059.2432

**Published:** 2022-12-20

**Authors:** Ling Xu, Noelle Fields, Alan Adolfo Kunz Lomelin, Kathryn Daniel, Daisha Cipher, Brooke Troutman

**Affiliations:** University of Texas at Arlington, Arlington, Texas, United States; The University of Texas at Arlington, Arlington, Texas, United States; University of Texas at Arlington, Arlington, Texas, United States; University of Texas at Arlington, Arlington, Texas, United States; The University of Texas at Arlington, Arlington, Texas, United States; Air Force Academy, Air Force Academy, Colorado, United States

## Abstract

To improve college students’ attitudes toward aging, an intergenerational intervention was implemented to connect them with community-dwelling older adults with cognitive impairment. College students were trained to make weekly phone calls to Meals on Wheels clients (age 65+). This study was an investigation of changes in college students’ attitudes toward aging over the course of study participation.College students aged 18 to 30 from a large public university in North Texas were recruited. Participants (n = 41) completed surveys through QuestionPro at baseline, midway, and at the end of the study. The Fraboni Scale of Ageism was used to measure attitudes toward aging. Friedman tests and one-way repeated measures ANOVAs were computed. Results indicated that college students’ total ageism scores significantly improved over time (F(2, 76)=4.491, p=.014), as well as their antilocution ageism scores (F(2, 76)=5.075, p=.007), and their avoidance ageism scores (F(2, 76)=3.844, p=.026). In addition, scores on six specific items significantly improved after participating in the study: “Many old people are stingy and hoard their money and possessions”, “Many old people are not interested in making new friends‚”, “Many old people just live in the past”, “I personally would not want to spend much time with an old person”, “Most old people should not be trusted to take care of infants”, and “Most old people would be considered to have poor personal hygiene”. Results suggest that weekly engagement with older adults offers promise for improving attitudes towards aging among college students. Implications for research and practice are discussed.

